# A stepped-wedge randomised controlled trial assessing the implementation, effectiveness and cost-consequences of the EDDIE+ hospital avoidance program in 12 residential aged care homes: study protocol

**DOI:** 10.1186/s12877-021-02294-8

**Published:** 2021-06-05

**Authors:** Hannah E. Carter, Xing J. Lee, Alison Farrington, Carla Shield, Nicholas Graves, Elizabeth V. Cyarto, Lynne Parkinson, Florin I. Oprescu, Claudia Meyer, Jeffrey Rowland, Trudy Dwyer, Gillian Harvey

**Affiliations:** 1 Australian Centre for Health Services Innovation and Centre for Healthcare Transformation, School of Public Health and Social Work, Faculty of Health, Queensland University of Technology, Kelvin Grove, 4059 Queensland Australia; 2 Duke-NUS Postgraduate Medical School, National University of Singapore, 8 College Rd, Singapore, 169857 Singapore; 3 Bolton Clarke Research Institute, 347 Burwood Hwy, Forest Hill, Victoria 3131 Australia; 4 Faculty of Health and Behavioural Sciences, The University of Queensland, Brisbane, QLD 4072 Australia; 5 Department of Psychiatry, University of Melbourne, Parkville, VIC 3010 Australia; 6 School of Medicine and Public Health, University of Newcastle, University Dr, Callaghan, NSW 2308 Australia; 7 School of Health and Behavioural Sciences, University of the Sunshine Coast, Sippy Downs, QLD 4556 Australia; 8 Rehabilitation, Ageing and Independent Living Research Centre, Monash University, Frankston, Victoria 3199 Australia; 9 Centre for Health Communication and Participation, La Trobe University, Bundoora, Victoria 3083 Australia; 10 Faculty of Medicine, University of Queensland, 20 Weightman St, Herston, QLD 4006 Australia; 11 Faculty of Health, School of Nursing, Kelvin Grove Campus, Queensland University of Technology, Brisbane, Australia; 12 Metro North Health, Royal Brisbane and Women’s Hospital, 7 Butterfield St, Herston, QLD 4029 Australia; 13 School of Nursing, Midwifery and Social Sciences, Central Queensland University, Rockhampton, QLD 4702 Australia; 14 College of Nursing and Health Sciences, Flinders University, Bedford Park, Australia 5042

**Keywords:** Residential aged care facility, Nursing home, Early detection, Deterioration, Acute care, Economic evaluation, High value care, Elderly, Hospital transfer, Emergency department

## Abstract

**Background:**

Older people living in residential aged care homes experience frequent emergency transfers to hospital. These events are associated with risks of hospital acquired complications and invasive treatments or interventions. Evidence suggests that some hospital transfers may be unnecessary or avoidable. The Early Detection of Deterioration in Elderly residents (EDDIE) program is a multi-component intervention aimed at reducing unnecessary hospital admissions from residential aged care homes by empowering nursing and care staff to detect and manage early signs of resident deterioration. This study aims to implement and evaluate the program in a multi-site randomised study in Queensland, Australia.

**Methods:**

A stepped-wedge randomised controlled trial will be conducted at 12 residential aged care homes over 58 weeks. The program has four components: education and training, decision support tools, diagnostic equipment, and implementation facilitation with clinical systems support. The integrated Promoting Action on Research Implementation in Health Services (i-PARIHS) framework will be used to guide the program implementation and process evaluation. The primary outcome measure will be the number of hospital bed days used by residents, with secondary outcomes assessing emergency department transfer rates, admission rates, length of stay, family awareness and experience, staff self-efficacy and costs of both implementation and health service use. A process evaluation will assess the extent and fidelity of program implementation, mechanisms of impact and the contextual barriers and enablers.

**Discussion:**

The intervention is expected to improve outcomes by reducing unnecessary hospital transfers. Fewer hospital transfers and admissions will release resources for other patients with potentially greater needs. Residential aged care home staff might benefit from feelings of empowerment in their ability to proactively manage early signs of resident deterioration. The process evaluation will be useful for supporting wider implementation of this intervention and other similar initiatives.

**Trial registration:**

The trial is prospectively registered with the Australia New Zealand Clinical Trial Registry (ACTRN12620000507987, registered 23/04/2020).

**Supplementary Information:**

The online version contains supplementary material available at 10.1186/s12877-021-02294-8.

## Background

In Australia, residential aged care (RAC) homes provide care and accommodation for older people who can no longer be supported to live in the community. Also referred to as residential aged care facilities or nursing homes, they involve the provision of daily personal care to residents as well as clinical support from qualified nursing staff. More than 200,000 Australians currently live in RAC homes [1].

Many residents are frail and their priority for medical care is good management of escalating comorbidities [2]. Yet, residents are often transferred to hospital after experiencing an acute deterioration in health [2, 3]. Previous studies have shown that up to one third of admissions in this cohort are potentially preventable [4–6]. A recent report from the Australian Medical Association estimated that there were over 27,000 potentially preventable hospital admissions from RAC homes in 2020–21, which translated to approximately 160,000 patient bed days and $AU 312 million in hospital costs [7]. In addition to unnecessary hospital admissions, residents may be transferred to emergency departments (EDs) for relatively minor conditions and then returned to the RAC home without admission. There were an estimated 49,000 of these non-admitted ED presentations from RAC homes in 2020–21, accounting for $AU 112 million in transport and ED triage costs [7].

There are several reasons why avoiding unnecessary hospital transfers and admissions is an important goal. A systematic review of outcomes following emergency transfer to hospital for residents of RAC homes found they were associated with high rates of in-hospital complications, with up to 80% of residents experiencing potentially invasive interventions and up to 34% dying in hospital [8]. There is evidence that hospital transfers are stressful for residents and their families, who prefer care to be provided in a familiar home environment [9]. Hospital admissions are also costly, and it is important to ensure they represent a high value use of resources.

The ‘Early Detection of Deterioration In Elderly residents’ or ‘EDDIE’ program is based on a model of care originally developed and piloted at one RAC home in Queensland, Australia [10]. The program aimed to prevent unnecessary hospital admissions by enhancing the ability of RAC home staff to respond appropriately to early signs of deterioration among residents. It involved provision of training and education, decision support tools, diagnostic equipment and tailored implementation strategies.

Evaluation of this pilot study found that EDDIE was feasible and well received [11]. Nursing staff reported feeling more confident and expressed a preference for managing residents within the RAC home, while personal care workers reported better collaboration with nursing staff [11]. The program reduced hospital transfer rates and average length of stay for residents admitted to hospital, resulting in a 41% reduction in total hospital bed days. A cost-effectiveness analysis found that the program had an 86% likelihood of being cost-effective [12].

An adapted version of the pilot program, named EDDIE+, has been developed to strengthen the successful elements of the pilot program and enhance the ability for the program to be scaled up, implemented, evaluated and sustained across a number of RAC homes. This study will implement EDDIE+ in 12 RAC homes operated by the study partner Bolton Clarke, a not-for-profit aged care provider. The program will adopt implementation science methods for embedding and sustaining change. The primary aim of the study is to reduce the number of hospital bed days used by RAC residents. The program will be evaluated using a Type 1 hybrid design [13] to simultaneously assess implementation, effectiveness, and health service outcomes.

## Methods and design

### Study design

**Fig. 1:**
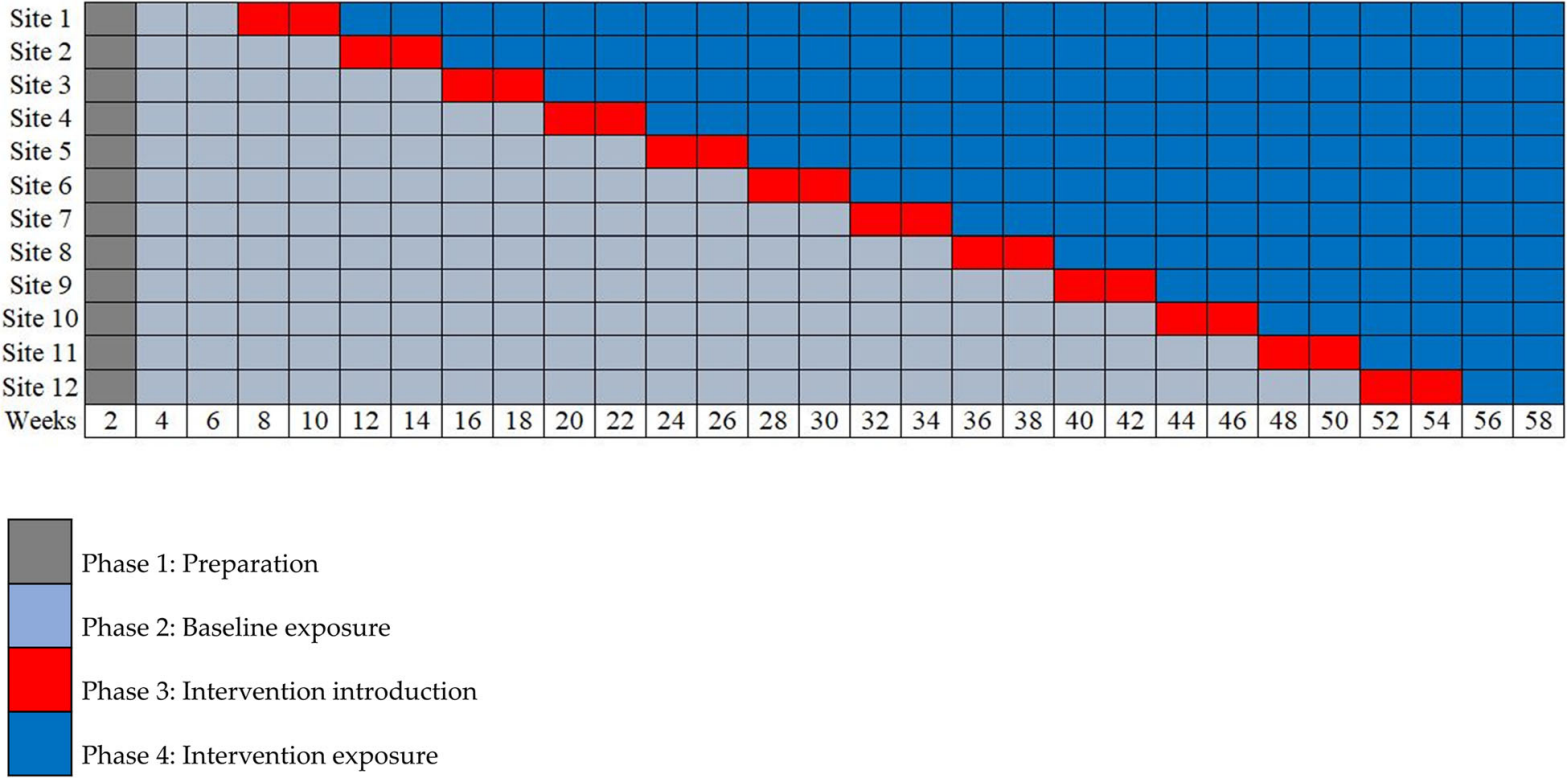
Stepped-wedge study design in 12 RAC homes

This study will adopt a stepped-wedge randomised controlled trial design (Fig. 1). Four phases will be sequentially rolled-out over 58-weeks: preparation; baseline (usual care) exposure; intervention introduction; and intervention exposure. The timing of the crossover from usual care to intervention introduction will be randomly allocated by the study statistician (XJL). Individual RAC homes will be notified of their intervention introduction date by the project team 10 weeks prior to commencement, to allow for adequate preparation time.

### Setting and participants

The trial will be undertaken at 12 RAC homes in Queensland, Australia. Data will be collected from five participant groups:

- RAC homes will be enrolled in the study as the main participant group. For inclusion, the RAC homes must be located in Queensland and be operated by the study partner, Bolton Clarke, a not-for-profit aged care provider. We will purposively sample from 21 eligible homes to ensure the program is implemented across a range of regional and metropolitan settings. A lower priority will be placed on homes with existing locally implemented hospital avoidance programs to minimise potential confounding with our study intervention. A lower priority will also be placed on homes with lower bed numbers to minimise the risk of underpowering the study.
- All nursing staff and personal care workers at each enrolled RAC home will participate in the study, with no exclusion criteria.
- Data on all residents at each enrolled RAC home will be collected as part of the study, with no exclusion criteria. A waiver of consent has been granted for resident data collection in recognition of the low privacy risk and negligible participant burden, as data will be de-identified and obtained indirectly from routinely collected databases and chart notes.
- All family members or nominated advocates of residents at each enrolled RAC home will be invited to participate in the study, with no exclusion criteria.
- A purposive sample of key internal and external stakeholders at each enrolled RAC home will be invited to participate in the study, with no exclusion criteria.

### Intervention

The EDDIE+ program is a multi-component intervention focused on improving quality of care. It aims to educate, engage and empower RAC home staff to identify early signs of deterioration in residents and proactively intervene to avoid unnecessary hospital transfers and admissions. An implementation science-based approach is embedded within the program to support system, process and staff behaviour change with the aim of enhancing the acceptability and sustainability of the program.

**Fig. 2:**
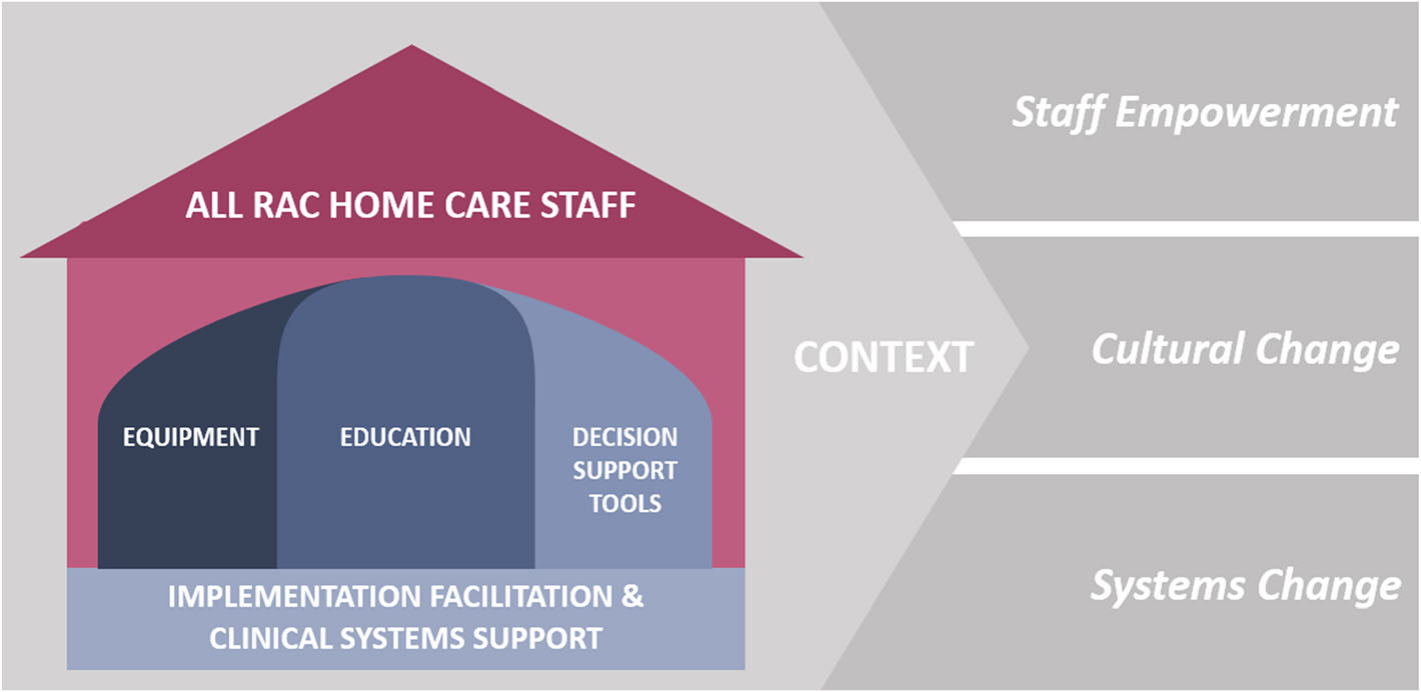
EDDIE+ intervention components and mechanisms of impact

EDDIE+ comprises four core components that can be tailored to meet the needs of the local context. These are: staff education and training; decision support tools; diagnostic medical equipment; and implementation facilitation (Fig. 2). The program adopts a holistic approach that seeks to engage all nurses and personal care workers within the home. It is expected that improvements from each of the core elements working synergistically will lead to sustained change via key mechanisms of impact including staff empowerment, cultural change and systems change. An intervention logic model outlining important contextual factors and mechanisms of impact is included in Additional file 1.

### Core components

The key activities to be included within each of the four core components have been defined, with varying levels of flexibility in their implementation, as follows:

#### Education and training of all nursing staff and personal care workers

Initial face-to-face training will be provided to all nursing staff and personal care workers, with a focus on early identification of deterioration and response, including roles/responsibilities of each staff member. Training will be mandatory and will be delivered by a nurse educator employed as part of the EDDIE+ study. Separate content and training sessions will be provided to nursing staff and personal care workers to reflect the different roles and responsibilities required of these positions. The length, intensity, delivery methods and depth of content coverage of the training will be adapted to suit the needs of each site. An educational materials toolkit will be developed including core content that the nurse educator must cover for all sites, and additional materials for use as required.

#### Decision support tools

A core decision support tool covering clinical decision-making pathways for managing deterioration across a number of specific conditions (e.g., dyspnoea, chest pain, dehydration) will be developed. This will be made available in hard or electronic copy depending on the needs and preferences of the local sites. Optional use of observation charts and communication tools will be made available.

#### Diagnostic medical equipment

The study will provide equipment to each RAC home based on an initial needs assessment, to include bladder scanners, electrocardiogram machines, vital signs monitors and pulse oximeters. Appropriate use of the equipment will be covered in the training sessions and educational materials.

#### Implementation facilitation and support

Each site will identify an internal EDDIE+ facilitator. This person will be in an existing clinical leadership position in the RAC home. The EDDIE+ facilitator will dedicate up to 1 day per week for the duration of the intervention phase to the implementation, facilitation, and data monitoring activities. A facilitator guide will be developed as a resource to support dedicated EDDIE+ facilitators in their role. This will include information on the nature of project team support available throughout the project as well as a set of resources to help guide the facilitation. Examples include templates for documenting engagement activities and/or other data collection requirements. RAC home executive/management will provide ongoing support, including through leadership presence at initial training and regular ongoing communication. EDDIE+ facilitators will assist the project team with local General Practitioner (GP) practice engagement, in recognition of GPs as key decision makers in RAC home hospital transfers. EDDIE+ facilitators will assist the project team with family engagement in recognition of the important role families play in resident care decisions.

### Implementation process and framework

EDDIE+ will adopt the integrated Promoting Action on Research Implementation in Health Services (i-PARIHS) framework to guide implementation and process evaluation. i-PARIHS is a widely used implementation framework which recognises the critical role of facilitation in assessing, aligning and integrating key implementation constructs [14]. Specific constructs defined within the framework that can act as barriers or enablers of implementation include the characteristics of the innovation to be implemented, the response of intended recipients of the intervention and contextual factors (across local, organisational and external levels).

The implementation of EDDIE+ will involve an initial environmental scan of recent and current hospital avoidance programs in place within Queensland RAC homes, and subsequent identification of adoption by Bolton Clarke homes. A more detailed context mapping exercise will then be conducted for each of the 12 enrolled RAC homes to generate a baseline context assessment against the i-PARIHS framework [15]. This process will determine readiness for change and potential enablers and barriers to change, such as the level of support among RAC home management and local GPs. A tailored implementation plan will be developed for each site based on the context mapping exercise. The implementation plan will detail the fixed and flexible elements to be adopted within each of the four core components, to support and monitor intervention fidelity.

### Monitoring and evaluating implementation

Process evaluation is an essential part of designing and testing complex interventions [16]. The real-world setting and length of this trial will require a pragmatic approach to intervention adherence, reach and fidelity. The project team, with the assistance of the local EDDIE+ facilitators, will systematically monitor the implementation of the program as part of the process evaluation, using templates and approaches based on the i-PARIHS constructs. This embedded approach will aim to provide direct support for implementing the program and will inform understanding regarding how the actual implementation process contributed to the study outcomes.

To ensure the innovation maintains relevance and is responsive to the diverse RAC home staff and contexts, this implementation phase will be an iterative cycle of Plan, Do, Study, Act [17]. Intervention fidelity of the program will be monitored during the iterative evaluation process. The EDDIE+ program will be mapped to the ‘Template for Intervention Description and Replication’ (TIDieR) checklist and guide [18] to promote the replicability of this research.

### Outcomes

The study outcomes and associated outcome measures are described in Table 1. They encompass measures of program effectiveness, cost consequences and implementation process.

**Table 1:** EDDIE + program outcome measures

Outcome label	Outcome	Outcome measures
**Effectiveness outcomes**		
Primary outcome (Outcome 1)	Number of hospital bed days	Total number of hospital bed days residents accrued during the baseline and intervention exposure periods, accounting for RAC home occupancy levels.
Outcome 2	Emergency Department (ED) transfer rates	Resident transfers to emergency departments including mode of transfer, diagnosis code, triage category, length of stay (mins) and discharge outcome.
Outcome 3	Hospital admission rates	Residents admitted to hospital as an inpatient (exclusive of dialysis admissions) including diagnosis code (Australian Refined Diagnosis Related Group (AR-DRG)) and mode of arrival.
Outcome 4	Length of hospital stay and discharge outcome	Time (in days/hours) from admission to discharge for each episode of care including diagnosis code, time in intensive care unit and discharge outcomes.
Outcome 5	Family awareness and experience	Family or nominated advocate responses to a short questionnaire and/or interview to ascertain awareness of EDDIE+ and family perceptions and experiences of the program.
Outcome 6	Staff self-efficacy	Staff scores on the Job-related and Group-related Self-Efficacy measure [19]
**Cost outcomes**		
Outcome 7	Cost of EDDIE+ implementation	The cost of implementing the EDDIE+ program will be measured by assigning profession specific wage rates, plus on-costs, to the duration of staff time associated with completing program activities (e.g. training, monitoring, facilitation). It will also include the costs of travel to sites, training materials and medical equipment.
Outcome 8	Cost of health service use	Health service costs to include ambulance transfers, ED presentations and hospital admissions.
**Process outcomes**		
Extent and fidelity of intervention (EDDIE+ program) implementation, impact, and contextual barriers and enablers of the EDDIE+ program		

### Data collection methods

#### Resident demographic and clinical data

Non-identifiable individual resident data at each RAC home will be extracted from the aged care provider’s existing routinely collected datasets. These will be used to report on the demographic characteristic summaries of the cohort to inform the generalisability of our results to other settings nationally and internationally. To review for seasonality and potential confounders over time we will use historical routinely collected data for residents at enrolled RAC homes for the two-year period directly before the trial start date.

#### Health services use and cost data

Non-identifiable individual resident data on transfer to and from EDs and admissions to hospital will be collected prospectively by the EDDIE+ facilitators based on resident care notes, with support from the project team. Data on monthly RAC home bed availability and occupancy rates will be used in calculating ED transfer and hospital admission rates.

To supplement the prospectively collected hospital transfer data, non-identifiable individual resident health service use and cost data will be retrospectively linked using state-level administrative datasets containing detailed information on ED and hospital diagnosis codes, length of stay, discharge outcomes and costs. These administrative data will be extracted using a data linkage process based on RAC home addresses for the dates of the trial period, as well as for a historical period of 2 years directly before the trial start date. Linked data will be validated against the prospectively collected ED and hospital transfer data from the enrolled RAC homes to ensure its accuracy.

#### Family awareness and experience survey activities

All current family members or nominated advocates of residents at enrolled sites will be invited to complete a short questionnaire on their awareness and experience of EDDIE+ at the end of the trial period. In addition, semi-structured interviews will be conducted with a purposive sample of family members or nominated advocates of residents both with and without an experience of hospital admission or proactive management of deterioration in the RAC home during the intervention exposure period. Interviews will be conducted until thematic saturation has been reached, up to a maximum of 30 interviews across the 12 sites.

#### Staff self-efficacy questionnaire

All nursing staff and personal care workers staff will be invited to complete a baseline measure of occupational self-efficacy using a validated questionnaire [19] to be administered immediately prior to staff training sessions. The self-efficacy questionnaire will be repeated in the final 2 weeks of the intervention exposure phase and up to 2 weeks post implementation.

#### Process evaluation

Process evaluation data will be collected using a series of templates based on i-PARIHS to guide the assessment of contextual barriers and enablers, conduct qualitative interviews with key stakeholders and undertake systematic implementation planning and record keeping.

Interview questions will focus on how the EDDIE+ program was introduced, any adaptations that were made, how staff responded, what changes in practice were implemented, and contextual factors that influenced implementation. Individuals from the following two stakeholder groups will be invited to voluntarily participate in a 30-min group or individual interview based on the i-PARIHS constructs: RAC home nursing staff and personal care workers; and, other RAC home stakeholders such as RAC home managers, EDDIE+ facilitators, Bolton Clarke executive management and health professionals who provide care to residents.

For each group, the participants will be purposively sampled to cover a representative cross section of stakeholder positions based on roles or interactions within the sites. Interviews will be conducted until thematic saturation has been reached, up to a maximum of 30 interviews for each stakeholder group across the 12 sites. Interviews will be conducted in the later stages of the implementation exposure phase up to 4 weeks post-trial.

### Data management

All research data will be stored on computer hard disk drives. These computers are networked to a password protected file storage server. The server has an automated daily batch back up procedure of the entire hard disk drive of each computer. Data will be shared via a password protected file storage server at the university leading the research that only members of the university-based project and investigator team can access. Data will be retained for a minimum of 15 years [20]. At the end of the study, final non-identifiable data sets will be deposited in a university Research Data Storage System. In line with publication embargoes and requirements, we will generate a document object identifier (DOI) for each non-identifiable data set and make these records publicly accessible.

### Sample size calculation

A simulation-based statistical power calculation was performed for the primary outcome of total hospital bed days. The simulations used a stepped-wedge design of 12 sites of 100 residents per site with 1 site switched from baseline phase to intervention exposure phase each month as shown in Fig. 1. The study design was estimated to have a 91% power to detect a 41% reduction in total bed days from a baseline period total hospital bed days of 346 bed days per 100 residents per year [12]. The power calculation was based on a 5% statistical significance level.

### Analysis

The time unit used in the analyses of Outcomes 1 to 4 is months. Data from the intervention introduction phase (phase 3) will not be in included in the statistical analyses but will instead inform the process evaluation. Subgroup analyses of Outcomes 1 to 6 will be performed for each RAC home separately. An initial analysis will be created using a scrambled intervention group by randomly allocating each RAC home to the baseline or intervention exposure period. A complete statistical report will be created using this scrambled data and sent to all investigators for discussion. This will allow investigators to query the methods and approaches used prior to the final report. It can also uncover errors in the code or data. Changes can be made prior to seeing the main results, which will help avoid the bias of only making changes where results are perceived as unfavourable.

There is a possibility that a disease outbreak (including COVID-19) could occur in one or more of the enrolled RAC homes during the study period. This may impact on hospital transfers and admissions and could potentially bias primary and secondary outcome findings. The analysis of Outcomes 1, 2, 3, 4 and 8 will therefore be conducted both with and without the inclusion of any outbreak related transfers and admissions.

### Analysis of primary outcome: Total number of hospital bed days

The primary outcome will be analysed using a mixed-effects Gamma regression model to estimate the impact of the EDDIE+ intervention while simultaneously accounting for RAC facility-specific variations in total hospital bed days. The key covariate will be the temporal indicator for the switch from baseline phase to intervention exposure phase, and the associated regression coefficient quantifies the change in total bed days associated with the EDDIE+ intervention period across all 12 RAC facilities. A linear time covariate in months since start of trial will be included in the regression model to capture any time trends in bed days that are not directly attributed to the intervention.

The regression model will also estimate RAC facility-specific random deviations from the overall baseline phase total hospital bed days from an RAC facility to account for any underlying differences at the different sites. It is possible that these random deviations are close to zero and the model fitting procedure may not converge. If this occurs, we will leave out the random intercept component and fit a standard Gamma regression model to the data.

#### Analysis of secondary outcomes

Outcomes 2 and 3 are rates of ED transfers and hospital admissions and will be investigated using a mixed-effects Poisson regression model. The offset or exposure variable will be the number of resident days, and outcomes expressed as rates per 1000 resident days. The covariates included will be a temporal indicator for the baseline phase to the intervention exposure phase switch to estimate the change associated with the EDDIE+ intervention, calendar time in months, mode of arrival, and random intercept term for each RAC facility.

Outcome 4 is admitted patient length of stay and will be analysed using a mixed-effects Gamma regression model. The covariates included will be a temporal indicator for the baseline phase to the intervention exposure phase switch to estimate the change in average length of hospital stay associated with the EDDIE+ intervention period, calendar time in months, resident age, resident sex, mode of arrival, and random deviations for each RAC facility from the overall pre-intervention average length of hospital stay.

Outcome 5 is family awareness and experience of EDDIE+ and will be investigated using a short questionnaire (Additional file 2) as well as semi-structured qualitative interviews conducted at the end of the trial period. Questionnaire responses will be summarised using descriptive statistics. Interview transcripts will be analysed and reviewed by two experienced qualitative researchers using NVivo software, and an inductive thematic analysis approach [21]. Concurrent analysis of interviews will iteratively inform subsequent interviews until saturation [22]. Illustrative quotes will be used to support the themes generated.

Outcome 6 is staff self-efficacy and will be measured through a validated questionnaire [19] administered to all nursing staff and personal care workers via a questionnaire at the commencement of the intervention introduction phase and at the end of the trial period. Separate analyses will be performed for the job-related self-efficacy questions and group-related self-efficacy questions. Internal consistency of the questionnaire will be assessed using the appropriate test statistic as determined by the distributions of the questionnaire items and total scores [23]. Difference between the average scores in the baseline and intervention exposure phases will be assessed using a *t*-test to obtain an overall change in staff’s self-efficacy before and after the intervention. Additionally, we will perform a linear regression analysis to investigate the potential contribution of different staff-related factors in the change in job-related self-efficacy average scores reported by staff between the baseline and intervention exposure phases. Relevant staff-related factors might include age, sex, staff role, years of experience in working in RAC facility, and RAC facility.

Outcome 7 is the cost of implementing the intervention and will be measured by valuing the costs of any materials, equipment and activities required as part of the intervention introduction and exposure phases. The duration of RAC home and project staff time associated with implementation activities, including training and attending meetings or other program-related activities, will be collected using prospective weekly activity logs completed by the EDDIE+ Nurse Educator and local EDDIE+ facilitators, to record minutes of staff time and grade of nursing staff and personal care workers. The economic opportunity costs of staff time will be valued using relevant wage rates plus on-costs to account for the full costs of employment. Quantities and types of consumables and incidentals used will be recorded and valued in monetary terms using market prices. The key outcome will be the estimated total cost of implementing the EDDIE+ intervention. Uncertainty will be represented using bootstrapped 95% confidence intervals.

Cost data on the health services used by residents during the trial will be retrieved from a state-level hospital costing database. These data will provide direct and overhead costs for each resident ED presentation and hospital admission. Statistical distributions will be used to describe variability in all cost items. The normal, uniform, beta and gamma distributions will be used depending on the type of parameter. Fitted distributions will be randomly re-sampled and the economic outcome of ‘change to total costs’ (outcome 8) simulated 10,000 times. This approach propagates uncertainty in prior parameters forward to the total cost outcomes. The key output will be the average cost per resident together with 95% bootstrap confidence intervals to estimate the uncertainty in this average.

Further details on planned residual checks and sensitivity analyses are included in Additional file 3.

#### Process evaluation data analysis

Interview notes and transcripts, combined with monitoring and field records, will be analysed initially by two experienced qualitative researchers applying i-PARIHS as an analytic framework [21]. Data that do not map to the i-PARIHS constructs will be subject to an inductive thematic analysis approach. Analyses will be iterative: firstly, identifying emerging themes, then comparing and refining these. Analysis will continue until no new themes emerge and agreement is reached [22].

#### Software

We plan to use R for data management, statistical analysis, and graphics of the quantitative analyses [24]. We plan to make all our R code publicly available via GitHub or a similar coding site. NVivo software will be used to support qualitative data analysis.

### Ethics and dissemination

Full ethical approval for this study has been granted by the Bolton Clarke Human Research Ethics Committee (approval number: 170031) with administrative ethical approval granted by the Queensland University of Technology University Human Research Ethics Committee (approval number: 2000000618).

A waiver of consent has been granted for access to non-identifiable resident demographic, clinical and health service use data. Nursing staff and personal care workers who complete self-efficacy questionnaires will imply consent by return of completed questionnaire, as will family members who complete the awareness and experience questionnaire. All RAC home staff members, family members and other stakeholders who agree to participate in group or individual interviews will be asked to provide written consent prior to commencing the interview.

Results from the study will be presented at conferences and published in peer-reviewed journal articles, adhering to the International Committee of Medical Journal Editors guidelines for authorship. Additionally, results will be disseminated to each participating RAC home through a series of presentations, reports and summaries. Key findings will be directly disseminated to our policy partners for further distribution to consumers, policy- and decision-makers in the form of evidence briefs, plain language summaries and policy recommendations.

## Discussion

The EDDIE+ program is focussed on upskilling nursing staff and personal care workers within RAC homes so they can better recognise and manage early signs of deterioration in residents, with the aim of reducing unnecessary hospital admissions. Building upon previous pilot work, it will provide training, decision support tools, facilitation support and medical equipment with the aim of empowering staff to deliver safe and effective care within the residents’ home environment. The program is grounded in the principles of implementation science, with an emphasis on creating system-level and cultural change to support the program becoming embedded in business-as-usual practice.

The avoidance of unnecessary hospital admissions is expected to improve resident outcomes by reducing the stress of hospital transfers for residents and families, while also preventing the risk of hospital acquired complications. Reducing unnecessary hospital transfers and admissions would free up resources for patients with greater health care needs and may reduce net health system costs. RAC home staff are also expected to benefit from increased feelings of empowerment in their ability to proactively identify and manage early signs of resident deterioration.

The stepped-wedge study design has several strengths. The incremental roll-out of the program to one site at a time is practical to implement, mimics how the intervention might be implemented in practice across other RAC providers [25] and is well-suited to the evaluation of health service delivery interventions [26]. This design allows the nurse educator and broader study team to work closely with each RAC home during the intervention introduction phase.

Each RAC home contributes data to both the baseline and intervention exposure groups, mitigating risk associated with comparing heterogeneous settings. This crossover also means that temporal effects can be studied [27] with more efficiency than other cluster designs [28]. The main drawbacks of this design are the potential risk of secular trends unrelated to the intervention exposure, and risk of unequal exposure to seasonal trends. This could include exposure to a widespread outbreak, for example COVID-19, or the implementation of other hospital avoidance initiatives. These risks were taken into consideration in the statistical analysis plan.

The study outcomes measures are focussed on demonstrating the avoidance of ED presentations and hospital admissions as proxy measures for improved resident outcomes. This is supported by a large body of evidence to suggest that hospital admissions towards the end of life may involve non-beneficial or potentially inappropriate care [29], elevated risk of hospital acquired complications, and poor prognostic outcomes [8, 30]. Collection of resident-reported outcome measures, such as quality of life, is not feasible in the context of this study. We determined that mortality rates would not be an appropriate measure of program effectiveness, given that the goals of end-of-life care may vary across individuals and may prioritise quality over duration of survival time [31].

Reducing unnecessary hospital transfers of aged care residents may benefit residents, families and staff, while also providing economic benefits for the health system. By concurrently completing an outcomes and process evaluation of the EDDIE+ program, we will identify the barriers and enablers to scaling up and implementing a multi-component hospital avoidance program within the RAC setting. This research will be useful for supporting further implementation of this intervention, and other similar initiatives.

## Supplementary Information

**Additional file 1.**

**Additional file 2.**

**Additional file 3.**

## Data Availability

At the end of the study, final non-identifiable data sets will be deposited in QUT’s Research Data Storage System. In line with publication embargoes and requirements, we will generate a document object identifier for each nonidentifiable data set and make this record publicly accessible.

## Abbreviations

AR-DRG: Australian Refined Diagnosis Related Group
EDDIE: Early Detection of Deterioration in Elderly residents
ED: Emergency Departments
GP: General Practitioner
i-PARIHS: Integrated Promoting Action on Implementation Research in Health Services
RAC: Residential aged care
TIDieR: Template for Intervention Description and Replication checklist
