# Supplementary figures and images for: A stepped-wedge randomised controlled trial assessing the implementation, effectiveness and cost-consequences of the EDDIE+ hospital avoidance program in 12 residential aged care homes: study protocol
Source: BMC Geriatr. 2021 Jun 5;21:347. doi: 10.1186/s12877-021-02294-8 (PMC8179705; doi:10.1186/s12877-021-02294-8)

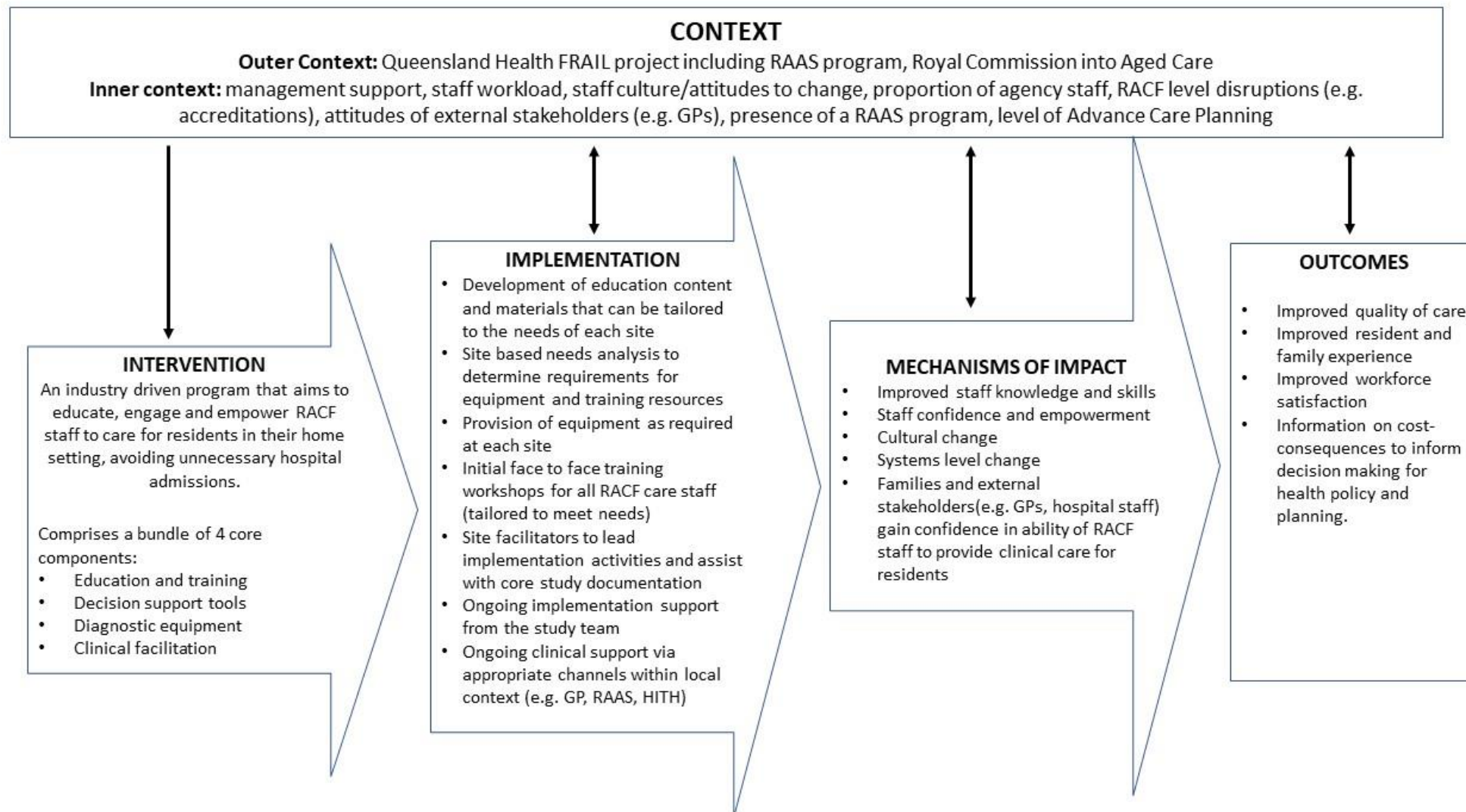

Supplement: Supplementary file 1 — Additional file 1. [file 12877_2021_2294_MOESM1_ESM.pdf]
