# Supplementary material for: A stepped-wedge randomised controlled trial assessing the implementation, effectiveness and cost-consequences of the EDDIE+ hospital avoidance program in 12 residential aged care homes: study protocol
Source: BMC Geriatr. 2021 Jun 5;21:347. doi: 10.1186/s12877-021-02294-8 (PMC8179705; doi:10.1186/s12877-021-02294-8)
Supplement: Supplementary file 2 — Additional file 2. [file 12877_2021_2294_MOESM2_ESM.pdf]

Researching Early Detection of Deterioration In Elderly residents

Family member or nominated advocate questionnaire

This survey asks your opinions about the EDDIE+ program at Bolton Clarke and how you feel it has affected the care your family member has received. There are no right or wrong answers to these questions.

Please circle the face that most reflects how you feels about the following statements.

1. How did you find your experience with the EDDIE+ program?

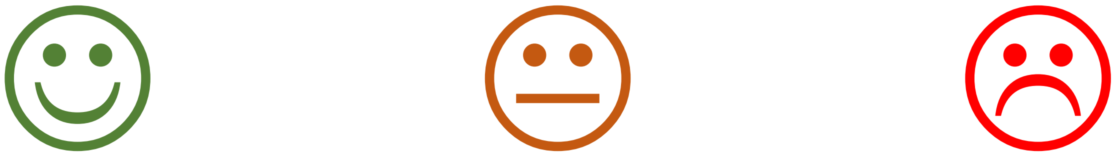

2. The EDDIE+ program impacted the care my loved one received in a good way.

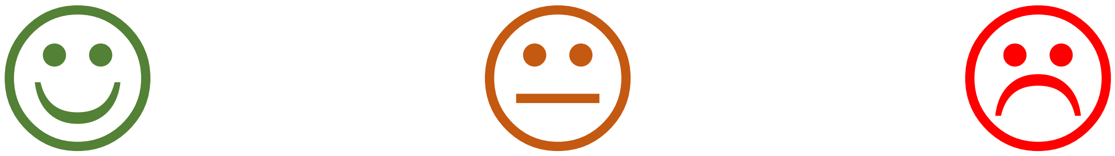

3. I think the EDDIE+ program should be introduced in other Residential Aged Care homes.

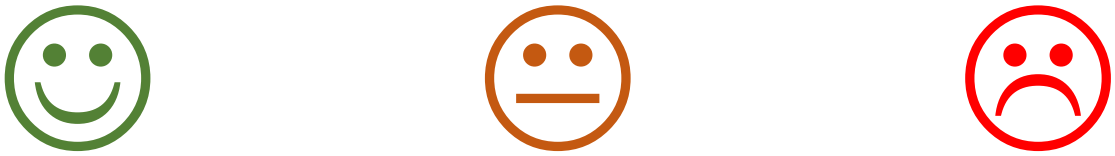

Thank you for completing this survey.
