# Supplementary material for: A stepped-wedge randomised controlled trial assessing the implementation, effectiveness and cost-consequences of the EDDIE+ hospital avoidance program in 12 residential aged care homes: study protocol
Source: BMC Geriatr. 2021 Jun 5;21:347. doi: 10.1186/s12877-021-02294-8 (PMC8179705; doi:10.1186/s12877-021-02294-8)
Supplement: Supplementary file 3 — Additional file 3. [file 12877_2021_2294_MOESM3_ESM.pdf]

Statistical methods supplement for the EDDIE+ protocol

This document outlines additional detail regarding historical data, residual checks and planned sensitivity analyses for the quantitative analysis of EDDIE+ outcomes data.

Historical data

If historical data on the primary outcome (hospital bed days) can be accurately extracted from administrative databases, these data will be reviewed prior to performing the proposed statistical analyses. We will investigate the historical data for potential seasonality and other time trends that should be incorporated into the statistical analyses. Additionally, we will compare historical data with baseline phase data for these outcomes to investigate if there was a notable Hawthorne effect from the trial and if it is possible to quantify the effect.

Residual checks

For all regression models, the residuals will be used to assess if the model: provides an adequate fit to the data, if the model assumptions are violated, and if there are any outlying or influential observations. These assessments include checking the distributional assumption of the models. For the Poisson regression model analyses of Outcomes 2 and 3, we will assess the fitted model for evidence of over-dispersion (i.e., violation of the Poisson distribution restriction). If detected, we will fit a negative binomial regression model instead which can account for the over-dispersion. Additionally, the data for Outcomes 2 and 3 will be checked for the number of zeroes as an exploratory data analysis step. If an excessive number of zeroes were detected (beyond what is expected of a Poisson or negative binomial distribution), we will fit a zero-inflated model variant of the regression models to account for the additional proportion of zero data observed. A zero-inflated model fits two regression models simultaneously: one for the count data (e.g., Poisson or negative binomial regression model), and another for the excess zero counts (e.g., Binomial logistic regression model).

Planned subgroup and sensitivity analyses

Subgroup analyses will be repeated for each RAC facility across all outcome measures. Results will be presented using the blinded RAC facility identifier.

For ED presentations (outcome 3), ED length of stay and discharge outcome will be additionally analysed using a competing risk, proportional hazards survival model with admitted as in-patient (including transfer to another hospital), discharged and death as competing endpoints for a resident's stay in ED in minutes. Cumulative incidence curves will be used to compare the event rates over time between the baseline and intervention exposure phases. The survival analysis will adjust for resident age, sex, mode of transfer and triage category.

The analysis plan for staff self-efficacy (outcome 6) assumes that it will not be possible to match most questionnaire respondents between the baseline and intervention exposure phases due to the voluntary nature of data collection. If at least 90% of the questionnaires

can be matched between the baseline and intervention exposure phases, the analysis plan will use statistical methods for paired data instead, i.e., paired *t*-tests and linear mixed regression model clustered by participant code and omit any unpaired data. Additionally, if it is determined that responses to individual questions in the questionnaire are strong indicators of self-efficacy individually, and needs to analysed separately, the analysis plan for outcome 6 will be modified to account for the ordinal nature of individual question's responses. Specifically, non-parametric tests like the Mann-Whitney U test will be used to compare question responses between the baseline and intervention exposure phases, and ordinal logistic regression analysis will be used in place of linear regression analysis.

Leave-one-out sensitivity analysis will be performed for outcomes 1 to 6, 7 and 8 by repeating the analysis multiple times, leaving out data from a different RAC facility in each repetition. The results from these analyses are then compared against the results obtained from the full data set to assess if data from a particular RAC facility was influential on the overall results.
